# Dynamic ^1^H imaging of hyperpolarized [1‐^13^C]lactate in vivo using a reverse INEPT experiment

**DOI:** 10.1002/mrm.26725

**Published:** 2017-05-05

**Authors:** Jiazheng Wang, Felix Kreis, Alan J. Wright, Richard L. Hesketh, Malcolm H. Levitt, Kevin M. Brindle

**Affiliations:** ^1^ Cancer Research UK Cambridge Institute University of Cambridge, Li Ka Shing Centre Cambridge United Kingdom; ^2^ School of Chemistry University of Southampton Southampton United Kingdom; ^3^ Department of Biochemistry University of Cambridge Cambridge United Kingdom

**Keywords:** hyperpolarization, imaging, pyruvate, lactate

## Abstract

**Purpose:**

Dynamic magnetic resonance spectroscopic imaging of hyperpolarized ^13^C‐labeled cell substrates has enabled the investigation of tissue metabolism in vivo. Currently observation of these hyperpolarized substrates is limited mainly to ^13^C detection. We describe here an imaging pulse sequence that enables proton observation by using polarization transfer from the hyperpolarized ^13^C nucleus to spin‐coupled protons.

**Methods:**

The pulse sequence transfers ^13^C hyperpolarization to ^1^H using a modified reverse insensitive nuclei enhanced by polarization transfer (INEPT) sequence that acquires a fully refocused echo. The resulting hyperpolarized ^1^H signal is acquired using a 2D echo‐planar trajectory. The efficiency of polarization transfer was investigated using simulations with and without T_1_ and T_2_ relaxation of both the ^1^H and ^13^C nuclei.

**Results:**

Simulations showed that ^1^H detection of the hyperpolarized ^13^C nucleus in lactate should increase significantly the signal‐to‐noise ratio when compared with direct ^13^C detection at 3T. However the advantage of ^1^H detection is expected to disappear at higher fields. Dynamic ^1^H images of hyperpolarized [1‐^13^C]lactate, with a spatial resolution of 1.25 × 1.25 mm^2^, were acquired from a phantom injected with hyperpolarized [1‐^13^C]lactate and from tumors in vivo following injection of hyperpolarized [1‐^13^C]pyruvate.

**Conclusions:**

The sequence allows ^1^H imaging of hyperpolarized ^13^C‐labeled substrates in vivo. Magn Reson Med 79:741–747, 2018. © 2017 The Authors Magnetic Resonance in Medicine published by Wiley Periodicals, Inc. on behalf of International Society for Magnetic Resonance in Medicine. This is an open access article under the terms of the Creative Commons Attribution License, which permits use, distribution and reproduction in any medium, provided the original work is properly cited.

## INTRODUCTION

**Figure 1:**
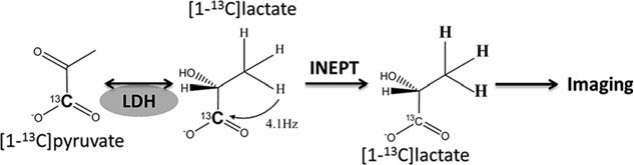
Lactate dehydrogenase catalyzes exchange of hyperpolarized ^13^C label between injected hyperpolarized [1‐^13^C]pyruvate and the endogenous lactate pool. Hyperpolarized [1‐^13^C]lactate is detected by transferring hyperpolarization from the C1 carbon to the spin‐coupled (J = 4.1 Hz) methyl protons in a reverse INEPT experiment.

The development of hyperpolarized ^13^C MRI using dynamic nuclear polarization of ^13^C‐labeled substrates has enabled imaging of metabolic fluxes in vivo 1, 2. ^13^C‐labeled pyruvate has been the most widely used substrate because it plays a central role in carbohydrate metabolism, it is relatively easy to polarize, and the long C1 carbon T_1_ (∼30 s in vivo), which makes the polarization relatively long lived, means that there can be substantial delivery and metabolism of the labeled pyruvate within the lifetime of the polarization 3. Imaging of hyperpolarized ^13^C‐labeled substrates requires an extra RF transmitter and receiver, in addition to the proton channel, and a more powerful gradient setup because the gyromagnetic ratio of ^13^C is one fourth that of ^1^H. In addition, the smaller gyromagnetic ratio of ^13^C means that the signal‐to‐noise ratio (SNR) is lower than for ^1^H and the transient nature of the ^13^C hyperpolarization means that signal averaging cannot compensate for this. Detection of hyperpolarized methyl protons in lactate would give, for the same level of polarization, a significant increase in SNR compared with direct detection of the ^13^C‐labeled C1 carbon. However, direct hyperpolarization of ^1^H is difficult due to its relatively short T_1_; the T_1_ of the lactate methyl protons in vivo at 4.7 T is about 1.7 s 4. Therefore, the feasibility of transferring nuclear spin polarization from the hyperpolarized ^13^C nucleus to ^1^H has been explored. Frydman and colleagues 5, 6 used a spatially encoded ultrafast Heteronuclear Single Quantum Correlation experiment for ^1^H detection of hyperpolarized ^13^C nuclei in high‐resolution solution experiments in vitro. Sarkar et al. 7 used a reverse insensitive nuclei enhanced by polarization transfer (INEPT) sequence for proton detection of hyperpolarized ^15^N choline, and Harris et al. 8 used a spatially selective variant of this experiment to monitor the kinetics of choline phosphorylation catalyzed by choline kinase in vitro. Recently, Dzien et al. 9 used a reverse INEPT sequence to study pyruvate decarboxylase activity in cultures of *S. cerevisiae* following injection of hyperpolarized [U‐^2^H_3_, 2‐^13^C]pyruvate. Chekmenev et al. 10 used a refocused INEPT sequence in spectroscopic studies in solution to transfer hyperpolarization from ^13^C to ^1^H in [1‐^13^C]succinate‐d_2_ and in 2,2,3,3‐tetrafluoropropyl 1‐^13^C‐propionate‐d_3_, and Truong et al. 11 used the same sequence, in conjunction with 2D fast steady state free precession ^1^H imaging, to image hyperpolarized 2‐hydroxyethyl‐^13^C‐propionate‐d_2,3,3_ in a phantom. Mishkovsky et al. 12 described spectroscopic studies in vivo, in which a heteronuclear polarization transfer sequence was used to acquire localized ^1^H spectra of hyperpolarized [1‐^13^C]acetate in rat brain, in which polarization was transferred from the carboxyl carbon to the methyl protons. We demonstrate here dynamic imaging of the conversion of hyperpolarized [1‐^13^C]pyruvate to lactate in tumor‐bearing mice in which labeled lactate in the tumor was detected via its methyl protons using a modified reverse INEPT experiment, in which a double dual‐spin echo sequence ensured acquisition of a fully refocused echo (Fig. 1).

## METHODS

### Transfer of Polarization from Lactate ^13^C_1_ to the Methyl Protons

We first discuss the use of a reverse INEPT sequence to transfer the longitudinal polarization of a single spin‐1/2 nucleus of isotopic species *S* to transverse polarization of a set of *N* magnetically equivalent spins‐1/2. The conventional reverse INEPT pulse sequence has the following form:

(1) $$90{\;}_{x}(S)-\tau_{1}/2-180{\;}_{x}(I,S)-\tau_{1}/2-90_{y}(I,S)-\tau_{2}/2-180_{x}(I,S)-\tau_{2}/2.$$

In the absence of relaxation, and assuming infinitely short pulses, the polarization transfer amplitude from the S‐spin to the *N* magnetically equivalent I‐spins is given by the functions 13, 14:

(2) $$f_{N}(\theta_{1},\theta_{2})={\left(cos\theta_{1}\right)}^{N-1}\sin\theta_{1}\sin\theta_{2},$$

where the delays are expressed as angles $\theta_{j}=\pi J_{CH}\tau_{j}$. If the initial ^13^C polarization level is denoted $p_{S}$, the maximum level of I‐spin polarization and the optimal values of the time variables are given by

(3) $$\begin{array}{c} \begin{array}{cc} N=1: & p_{1} \end{array}(\text{max})=p_{S}\;\;\text{at}\;\;\theta_{1}=\theta_{2}=90{}^{\circ}\\ \begin{array}{cc} N=2: & p_{1} \end{array}(\text{max})=p_{S}/2\;\;\text{at}\;\;\theta_{1}=45{}^{\circ},\theta_{2}=90{}^{\circ}\\ \begin{array}{cc} N=3: & p_{1} \end{array}(\text{max})=\frac{2p_{S}}{3\sqrt{3}}\;\;\text{at}\;\;\theta_{1}=\arctan\frac{1}{\sqrt{2}}≃35.3{}^{\circ},\theta_{2}=90{}^{\circ} \end{array}.$$

The case *N* = 1 is relevant to polarization transfer from lactate ^13^C_1_ to the C2 proton. In the best case, the initial ^13^C polarization level, *p*_*S*_, is preserved upon transfer to the C2 proton, leading to an enhancement in hyperpolarized magnetization by a factor $\gamma_{1}/\gamma_{S}≃3.97$, taking into account the relative gyromagnetic ratios. The case *N* = 3 is relevant to polarization transfer from lactate ^13^C_1_ to the methyl protons. In the best case, the methyl protons acquire a polarization of 0.385 *p*_*S*_. The hyperpolarized magnetization is therefore enhanced by the factor $3\times 0.385\times \gamma_{1}/\gamma_{S}≃4.59$, taking into account the number of polarized protons and the relative gyromagnetic ratios. Furthermore, optimal transfer to the methyl protons occurs at a much shorter $\tau_{1}$ interval, assuming equal J‐couplings. In fact, the coupling constant between the C1 carbon and the C3 methyl protons in [1‐^13^C]lactate is larger than the coupling constant with the C2 proton (3.2 Hz versus 4.1 Hz) 15. In the absence of relaxation, the optimal value of $\tau_{1}$ is therefore approximately three times shorter, and the achievable ^1^H magnetization 15% larger, when the methyl protons are targeted, compared to the C2 proton. Because short pulse sequence intervals generally lead to smaller relaxation losses, the lactate methyl protons are a more promising target for polarization transfer than the C2 proton.

### Pulse Sequence

**Figure 2:**
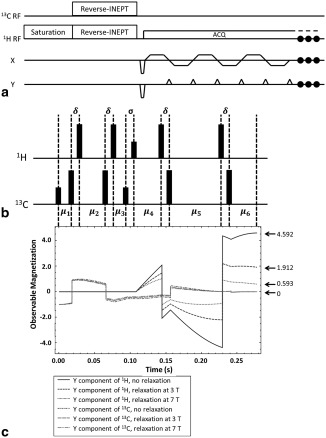
(A) Pulse sequence for transferring hyperpolarization from the C1 carbon to the methyl protons of lactate and imaging of the resulting hyperpolarized proton signal. (B) Pulse sequence for the double dual‐spin echo INEPT (reverse INEPT) module shown in panel A. Shorter and longer bars refer to 90 ° and 180 ° pulses, respectively. The 90 ° pulse on ^1^H is the ^1^H excitation pulse. The first and second 90 ° pulses on ^13^C are the ^13^C excitation and flip‐back pulses, respectively. The phases of the RF pulses are, in the order as displayed, x,x,x,x,x,y,−y,x,x,x,x. (C) Evolution of ^13^C and ^1^H *y* magnetizations in [1‐^13^C]lactate during the reverse INEPT module. Three simulations are shown with: no relaxation, ^1^H and ^13^C T_1_ and T_2_ relaxation at 3T, and ^1^H and ^13^C T_1_ and T_2_ relaxation at 7T. The *y* component of the methyl group proton magnetization is shown.

The pulse sequence (Fig. 2A) starts with a saturation module on the proton resonances, so that unwanted signals from water and lipids are suppressed, followed by a reverse INEPT preparation module, after which prephasing gradients are applied on both readout and phase encoding axes, followed by a symmetric echo‐planar acquisition train 16. The transmission coil in our setup could not be used to pulse simultaneously on ^1^H and ^13^C and therefore there was a delay between the ^1^H and ^13^C pulses, which otherwise would happen at the same time in a conventional INEPT sequence. In the modified INEPT preparation sequence, the ^1^H and ^13^C coherences evolve with the same phase as in a conventional INEPT sequence at each of the 90 ° pulses and at the end of the preparation period (Fig. 2B). If relaxation effects are neglected, maximum polarization transfer occurs when

(4) $$\{\begin{array}{c} \mu_{1}+\mu_{2}+\mu_{3}-2\delta =\text{acos}\left(\sqrt{\frac{2}{3}}\right)/\pi J=47.78\;ms\\ \mu_{4}+\mu_{5}+\mu_{6}-2\delta =\frac{1}{2J}=121.952\;ms \end{array},$$

where δ is the center‐to‐center delay between a pair of ^13^C and ^1^H pulses. In order for spin echoes to be formed at the time of the second ^13^C 90 ° pulse in the reverse INEPT module, when magnetization is flipped back along the z axis, and at the end of this module (at the end of $\mu_{6}$) (Fig. 2B), the timing must fulfill the following conditions:

(5) $$\{\begin{array}{c} \mu_{2}=\mu_{1}+\mu_{3}\\ \mu_{5}=\mu_{4}+\mu_{6} \end{array}.$$

Equations 2 and 3 determine the values of $\mu_{1},\mu_{2},\ldots \;\;,\mu_{6}$. The interval σ was kept to a minimum and was determined by the length of the ^13^C flip‐back and ^1^H excitation pulses. The total duration of the reverse INEPT module, from the first ^13^C pulse to the echo formed at the end of the module, was 278 ms.

The saturation module consisted of a 4‐ms 90° sinc pulse, with a bandwidth of 8 kHz, followed immediately by a spoiler gradient in the slice direction. A 4‐ms sinc‐shaped pulse was designed with the SLR algorithm 17 for both excitation and flip‐back of the ^13^C coherences (the first and second 90° pulses on ^13^C). The bandwidth was 600 Hz to allow selective excitation of [1‐^13^C]lactate without disrupting the [1‐^13^C]pyruvate polarization. A sinc‐shaped ^1^H pulse was used for selective excitation of the lactate C3 methyl protons (the 1^st^ 90° pulse on ^1^H). The bandwidth was 1500 Hz to avoid excitation of the C2 and water protons. The ^13^C and ^1^H magnetizations were inverted using 10 ms adiabatic hyperbolic‐secant pulses 18. The bandwidth of the ^13^C pulses was 8 kHz, so that even far off‐resonance [1‐^13^C]pyruvate magnetization (far from the magnet isocenter) would experience full inversion and the hyperpolarization would not be destroyed by the pulses. For the ^1^H pulses, the bandwidth was only 1000 Hz to avoid inversion of the C2 proton resonance (approximately 850 Hz from the C3 proton resonance).

The dual‐spin echo design was required because an adiabatic pulse results in a non‐linear phase change across the swept frequency range, which can only be cancelled by another adiabatic pulse with the same waveform and RF power 19, 20. This sequence also ensures that the spin echo resulting from phase evolution induced by local $B_{0}$ field variations coincides with complete polarization transfer, under conditions where the ^1^H and ^13^C pulses cannot be applied simultaneously.

### Simulation of the Effects of Relaxation

Evolution of the ^13^C and ^1^H polarizations during the reverse INEPT preparation block was simulated in the weak‐polarization limit using the SpinDynamica platform (available online at www.spindynamica.soton.ac.uk) in Wolfram Mathematica (version 10.4; Wolfram Research, Inc., Champaign, Illinois, USA). For simplicity, shaped pulses were treated as being infinitely short and relaxation losses during the pulses were neglected. A relaxation model of uncorrelated random fields was used.

### Spectroscopic Experiments with [1‐^13^C]Lactate at Thermal Equilibrium

The validity of these simulations was tested experimentally by implementing the reverse INEPT experiment on a high‐field (14.1T), high‐resolution NMR spectrometer (Bruker Spectrospin Ltd., Coventry, United Kingdom), where the higher sensitivity allowed observation of transfer of thermal ^13^C polarization into ^1^H. Experiments were performed with 1 M [1‐^13^C]lactate in 100% D_2_O using a 5‐mm ^1^H/broadand inverse detection probe (Bruker Spectrospin Ltd.). To eliminate signal originating from direct proton excitation, the pulse sequence was phase cycled, wherein alternate acquisitions the phases of the initial 90° pulse and the receiver were shifted by 180°. T_1_ relaxation times were measured with an inversion recovery sequence (n = 1, TR_1H_ = 25.6 s, TR_13C_ = 300 s). The time between the 90 and 180 pulse was varied between 0.2 s to 25.6 s for the ^1^H acquisitions and from 2.34 s to 300 s for the ^13^C acquisitions. T_2_ relaxation times were measured with a Carr‐Purcell‐Meiboom‐Gill sequence (n = 1, TR_1H_ = 15 s, TR_13C_ = 90 s). The minimum echo time was 10.054 ms and over 16 acquisitions the number of echoes was increased to 1000 (TE_max_ = 10.054 s).

### MR Scanner

Experiments were performed on a 7T Agilent scanner (Agilent, Palo Alto, California, USA) with a 42‐mm diameter ^1^H and ^13^C transmit/receive volume coil (Rapid Biomedical, Rimpar, Germany).

### Phantom Experiments

**Figure 3:**
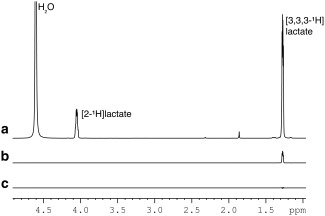
Polarization transfer from ^13^C to ^1^H in thermally polarized [1‐^13^C] lactate. (A) 90 ° pulse and acquire ^1^H spectrum. (B) Transfer of polarization from 1‐^13^C to 3,3,3‐^1^H_3_ using the reverse INEPT sequence. Phase cycling ensured that only transferred polarization was observed. (C) Spectrum acquired using the reverse INEPT sequence with no ^13^C pulses. All spectra are the sum of 32 transients. The reverse INEPT spectra were acquired with TR = 90 s to allow full ^13^C relaxation; the directly detected ^1^H spectrum was acquired with TR = 15 s to allow full ^1^H relaxation.

A 60‐µL [1‐^13^C]lactate sample containing 58 mg 50% wt/wt [1‐^13^C]lactate solution (Sigma‐Aldrich, St. Louis, Missouri, USA), 15 mM OXØ63 (GE Healthcare, Amersham, United Kingdom), 1.2 mM Dotarem gadoterate meglumine (Dotarem; Guerbet, Roissy, France), and 20 µL 1/10 vol/vol dimethyl sulfoxide (Sigma‐Aldrich) was hyperpolarized for 2 h using a Hypersense polarizer (Oxford Instruments, Abingdon, United Kingdom) at 1.2 K in a magnetic field of 3.35T with microwave irradiation at 94.116 GHz. The hyperpolarized sample was then dissolved in 4 mL superheated phosphate‐buffered saline, and 0.5 mL was injected into an 18‐mm inner diameter sphere filled with water. Two spectra were acquired using the pulse sequence shown in Figure 2, but without the imaging gradients (Fig. 3A). The delay between the INEPT preparation module and the beginning of signal acquisition was 170 ms, calculated from the center of the 90° ^1^H excitation pulse, which was set at the C3 ^1^H resonance frequency (Fig. 2B). Data were acquired into 2048 points covering a bandwidth of 12.5 kHz. In a second experiment, hyperpolarized [1‐^13^C]lactate was injected and a series of echo planar images were acquired from the C3 ^1^H resonance, with a time resolution of 2 s and starting 2 s after the completion of the lactate injection (a single image is shown in Fig. 3B). The receiver bandwidth was 125 kHz and the echo spacing 400 µs. A field of view of 4 × 4 cm^2^ covered a 32 × 32 data matrix, and the k‐space center was acquired after only four echoes to minimize the echo time (173 ms). A ^1^H fast spin echo image was acquired (256 × 256, 4 × 4 cm^2^, slice thickness 80 mm) to provide a positional reference.

### Tumor Model

Animal experiments were performed in compliance with a project license issued under the Animals (Scientific Procedures) Act of 1986. Protocols were approved by the Cancer Research UK, Cambridge Institute Animal Welfare and Ethical Review Body. EL4 lymphoma cells (5 × 10^5^) were injected subcutaneously into the lower flank of female C57BL/6J mice, and the resulting tumor was allowed to grow for 8 days, when it was >1 cm in diameter.

### Dynamic Imaging In Vivo

The mouse was fasted for 6 hours before imaging 21 and warmed at 32°C 1 h before induction of anesthesia using 1.5%–2.5% isoflurane. The [1‐^13^C]pyruvate sample contained 44 mg [1‐^13^C]pyruvic acid (CIL, Tewksbury, Massachusetts, USA), 15 mM OXØ63 and 1.4 mM Dotarem and was hyperpolarized using the Hypersense polarizer. Before injection, it was dissolved rapidly in 6 mL buffer containing 40 mM Tris, 185 mM NaOH, and 100 mg/L ethylenediaminetetraacetic acid heated to 180°C and pressurized to 10 bar. The injection took 8 s, and imaging started 10 s after completion of the injection when a substantial amount of [1‐^13^C]lactate had already been generated from the injected [1‐^13^C]pyruvate. Images were acquired every 2 s, and a total of 30 images were acquired, with the seventh acquisition used as a reference for echo planar imaging phase correction. Ninety‐degree pulses were used for ^13^C excitation and flip‐back so that all of the [1‐^13^C]lactate polarization produced from the injected [1‐^13^C]pyruvate during each 2 s interval was detected in the ^1^H image. A 90° flip angle was used for ^1^H excitation to make full use of the transferred polarization. The same acquisition parameters were used for in vivo and phantom imaging.

T_2_‐weighted proton fast‐spin echo images (16 slices, slice thickness = 2 mm) with a 128 × 128 data matrix covering a 4 × 4 cm^2^ field of view were acquired to provide a positional reference.

### Image Reconstruction

Phase correction was performed using the reference image data, as described by Zhou 16. The partial k‐space was then zero‐filled from 20 × 32 to 32 × 32 before Fourier Transformation. Phase correction and image reconstruction were performed in MATLAB (MathWorks, Natick, Massachusetts, USA).

## RESULTS

Hyperpolarized ^13^C label is exchanged between injected hyperpolarized [1‐^13^C]pyruvate and the endogenous unlabeled lactate pool in the reaction catalyzed by lactate dehydrogenase (Fig. 1A) 1. Polarization was transferred from the C1 carbon to the indirectly coupled C3 methyl protons (J = 4.1 Hz) using a reverse INEPT sequence (Fig. 2B) and the resulting hyperpolarized ^1^H signal imaged using an echo planar imaging readout (Fig. 2A). Simulations showed that evolution of the magnetization of the three magnetically equivalent methyl ^1^H spins in [1‐^13^C]lactate under the four‐spin coupling Hamiltonian, relative to the initial magnetization of the hyperpolarized ^13^C spin, enhances the hyperpolarized magnetization by a factor of 4.6 and that this is decreased by relaxation to a factor of 1.9 at 3T, and to 0.6 at 7T (Fig. 2C). The simulations that included relaxation were performed using the following published values for T_1_ and T_2_ at 3T and 7T: $T_{2}^{13C}$ (7 T) = 300 ms 22, $T_{2}^{13C}$ (3 T) = 520 ms 23, $T_{2}^{1H}$ (7 T) = 100 ms 24, $T_{2}^{1H}$ (3 T) = 256 ms 25, $T_{1}^{13C}$ (3T) = 45 s 26, and $T_{1}^{1H}$ (4.7T) = 1.73 s 4. These simulations were tested experimentally using thermally polarized 1M [1‐^13^C]lactate at a high field (14.1T). The T_1_ and T_2_ relaxation times of the 1‐^13^C and 3,3,3‐^1^H lactate resonances were measured using inversion recovery and CPMG sequences respectively, yielding $T_{1}^{1H}$ = 2.2 ± 0. 1 s, $T_{2}^{1H}$ = 1.6 ± 0.1 s, $T_{1}^{13C}$ = 15.8 ± 0.1 s, and $T_{2}^{13C}$ = 3.5 ± 0. 1 s. Simulation of the reverse INEPT experiment using these relaxation times yielded an enhancement of 0.082, which was in good agreement with a value of 0.084 measured experimentally (compare the methyl peak intensities in Fig. 3A and 3B).

**Figure 4:**
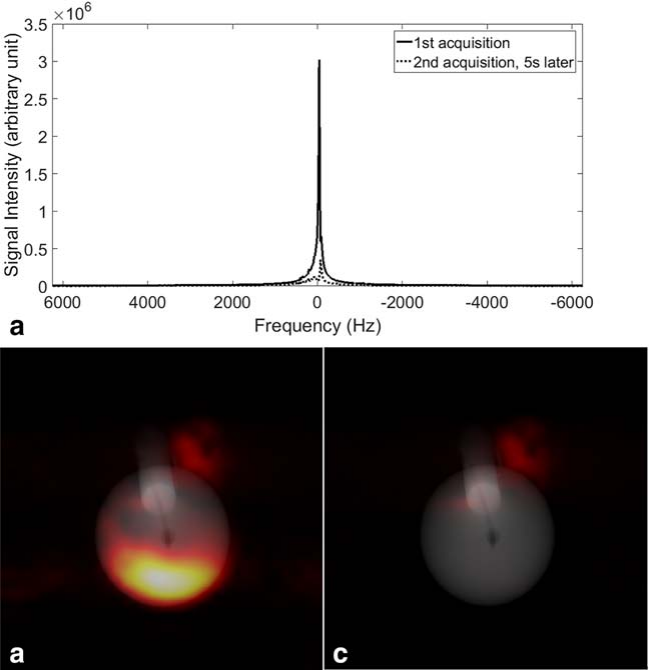
Phantom experiments with hyperpolarized [1‐^13^C]lactate. (A) ^1^H spectra acquired after injection of hyperpolarized [1‐^13^C]lactate into the phantom, where the ^1^H excitation was set to the methyl proton resonance frequency. The second spectrum (dotted line) was acquired 5 s later. (B, C) Sequential methyl group ^1^H images acquired after injection of hyperpolarized [1‐^13^C]lactate into the phantom. The lactate proton images, which are rendered in color, have been overlaid on a fast‐spin echo water ^1^H image, which has been rendered in grayscale.

^1^H spectra and images acquired using the reverse INEPT sequence, following injection of hyperpolarized [1‐^13^C]lactate into a phantom, are shown in Figure 4. ^1^H signal in the first acquisition (solid line in Fig. 4A and image shown in Fig. 4B) was approximately 10 times larger than in the second acquisition (dotted line in Fig. 4A and image shown in Fig. 4C) due to depletion of the ^13^C hyperpolarization by the 90° ^13^C excitation pulse. The methyl proton resonance had a peak width at half height of about 35 Hz and therefore splitting due to ^1^H and ^13^C coupling was not resolved (the methyl proton resonance of [1‐^13^C]lactate is split into a doublet by coupling to the C2 proton (J = 6.9 Hz) and these doublets are further split into doublets by coupling to the C1 ^13^C (J = 4.1 Hz). In the image, this splitting of the methyl proton resonance will not compromise SNR if the k‐space center is acquired at the time when the spin echo is formed, where the image signal is then the integral of all the in‐phase peaks. The SNR of the spectrum from the first acquisition was 8618, which decreased to 1560 for the second acquisition. The SNR for the first image was 586.4 and only 56.9 for the second. The spectrum and image SNRs were measured as the ratios between maximum and mean signals, respectively, and the standard deviation of the background noise 27. There was no observable excitation of the water resonance, which should be about 1090 Hz away from the lactate methyl proton resonance. The residual signal observed in the second image (Fig. 4C) appeared to be water signal from the injection line. The B_0_ field was only shimmed over the spherical phantom. Water protons in the injection line may therefore have been off‐resonance and excited by the transition band of the proton inversion pulses. The residual signal was spatially displaced from the injection line in the phase encoding direction, consistent with it being from off‐resonance signal. The hyperpolarized [1‐^13^C]lactate solution (0.5 mL) was injected into the bottom of the sphere phantom, which contained 3 mL of water, and the first acquisition started only 2 s after completion of the injection. The methyl proton signals were concentrated, therefore, at the bottom of the phantom.

**Figure 5:**
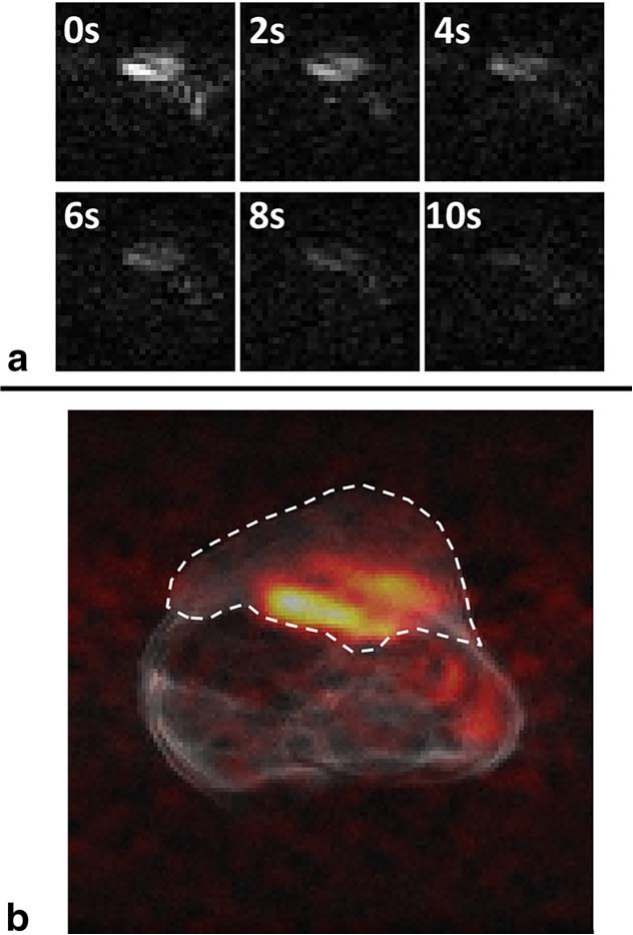
(A) Dynamic ^1^H images of the lactate methyl protons acquired using the reverse INEPT sequence at the indicated times following injection of hyperpolarized [1‐^13^C]pyruvate into a tumor‐bearing mouse. The first image (at 0 s) was acquired 2 s after completion of the injection, which took a total of 10 s. (B) The image in panel A acquired at 0 s and rendered in false color overlaid on a fast spin echo ^1^H image of tissue water, which is in grayscale. The tumor is outlined.

Dynamic images of the methyl proton resonance of hyperpolarized [1‐^13^C]lactate were acquired using the reverse INEPT sequence following injection of hyperpolarized [1‐^13^C]pyruvate into an EL4 tumor‐bearing mouse (Fig. 5A). A series of images are shown in Figure 5A and an overlay of the first image, rendered in false color, on an anatomic image acquired using a ^1^H fast spin echo sequence is shown in Figure 5B. The ^1^H signals from hyperpolarized [1‐^13^C]lactate were observed at the base of the tumor and adjacent to the body of the animal. We have observed a similar distribution of labeled lactate in this tumor model using direct ^13^C detection (data not shown). Unlike in the phantom, the hyperpolarized ^13^C and ^1^H signals in the tumor are sustained over time by inflow of hyperpolarized [1‐^13^C]pyruvate into the tumor from the rest of the animal.

## DISCUSSION

The reverse INEPT pulse sequence transfers ^13^C nuclear spin polarization in hyperpolarized [1‐^13^C]lactate from the ^13^C_1_ carbon to the spin‐coupled methyl protons. The SNR for the lactate ^1^H image acquired in vivo was 17 (Fig. 5B), which is comparable to SNR values obtained previously in this tumor model with direct ^13^C detection, where the SNR of ^13^C images of [1‐^13^C]lactate acquired using a 90° pulse and summed over a 20‐mm thick slab were between 13 and 20 28. However, these ^13^C images were acquired using a 20‐mm‐diameter surface coil placed around the tumor, whereas the ^1^H images shown here were acquired using a 42‐mm‐diameter ^1^H and ^13^C transmit/receive volume coil. Given the difficulty in ensuring equal coil efficiencies and to generalize the relevance of the measurements shown here for other field strengths, the experiment was simulated. Simulation using published values for the T_1_ and T_2_ of the ^1^H and ^13^C nuclei in lactate in vivo showed that transferring ^13^C hyperpolarization into the methyl protons enhances the hyperpolarized magnetization by a factor of 4.6, and that this is decreased by relaxation to 1.9 at 3T, and to 0.6 at 7T, which was the field strength used here. The amplitude of the detected NMR signal depends on this polarization but is also proportional to the precession frequency, because it is generated by electromagnetic induction in the receiver coil. Assuming identical coil efficiencies, then detecting spin polarization in ^1^H rather than ^13^C is beneficial due to the higher gyromagnetic ratio of the proton, γ_1H_; for a given level of polarization, signal increases as ∼γ^2^ 29 as magnetization is proportional to γ and, given the same magnetization, the current induced in the receiver coil is also proportional to γ. Hence, for the same level of polarization, ^1^H will generate a signal in the receiver coil that is approximately 16 times larger than that for ^13^C. With the simulated values for the magnetizations, which includes T_1_ and T_2_ relaxation of the ^13^C and ^1^H spins, detection of the ^13^C_1_ polarization via the methyl protons will increase the signal 7.6 fold at 3T and 2.4 fold at 7T. These numbers were obtained by multiplying the simulated magnetizations by four (the same operation was performed for all the SNR calculations shown below). However, noise also increases with frequency. Noise due to coil resistance, $R_{C}$, is proportional to the square root of the frequency of the induced alternating current whereas noise due to sample resistance, $R_{S}$, increases quadratically with frequency. Because the overall noise signal is proportional to $\sqrt{R}=\sqrt{R_{C}+R_{S}}$, the SNR that takes account of sample and coil noise can be calculated as 30

(6) $$SNR=\frac{v^{2}}{{\left[\alpha a^{2}v^{1/2}+\beta v^{2}b^{5}\right]}^{1/2}},$$

where v is the Larmor frequency, *a* and *b* are coil geometry parameters, and *α* and *β* are weightings for the two sources of noise, where *α* represents coil noise and *β* sample noise. Assuming the same coil geometry, the SNR for ^1^H is 11.3 ( $16/\sqrt{2}$) times that for ^13^C when sample noise is neglected (*α* = 1, *β* = 0). With the calculated ^13^C and ^1^H magnetizations this will give an SNR benefit when detecting ^13^C hyperpolarization via the methyl protons of 5.4 times at 3T and 1.7 times at 7T (magnetization enhancement × $4\sqrt{2}$, where the denominator is determined by coil noise, which is proportional to the fourth root of the Larmor frequency, as shown in Equation 6). If sample noise dominates (*α* = 0, *β* = 1) detection via ^1^H gives an SNR benefit of only 4 times that of ^13^C detection if relaxation is ignored and, given the calculated ^13^C and ^1^H magnetizations, the SNR benefit would decrease to 1.9 times at 3T and 0.6 times at 7T (4 × magnetization enhancement/4, where the denominator comes from the fact that sample noise is proportional to the Larmor frequency, as shown in Eq. 6). Although sample noise is thought to dominate with relatively large imaging objects at high magnetic fields 31, this is evidently not the only source of noise, because superconducting coils show an increased ^1^H SNR of 2‐ to 5‐fold at fields between 1.5T and 9.4T when compared with room temperature copper coils 32, 33, 34. Therefore, even at 7T, there may still be a SNR benefit in detecting hyperpolarized [1‐^13^C]lactate via the spin‐coupled methyl protons. There was some evidence for this in the measurements made here.

The dynamic images (Fig. 5A) showed rapid signal decay as each acquisition sampled effectively all of the hyperpolarized signal from [1‐^13^C]lactate generated from hyperpolarized [1‐^13^C]pyruvate in the preceding 2 s. Such a rapid decay has been observed previously in saturation‐recovery experiments, where following injection of hyperpolarized [1‐^13^C]pyruvate the [1‐^13^C]lactate produced was sampled with repeated spectrally selective 90° ^13^C pulses 35. This problem could be addressed by using a preparation module that allows partial transfer of the polarization 36. This would also allow serial observations of the pyruvate resonance, which is not possible with the reverse INEPT sequence, because all of the polarization is effectively transferred following the first application of the pulse sequence. The simulation shown in Figure 2C shows that there is also the potential for shortening the reverse INEPT module, and thus reducing signal loss due to T_2_ decay, because this may be effected without significantly reducing polarization transfer.

The longer ^1^H and ^13^C T_2_ relaxation times at lower magnetic field strengths will improve the efficiency of polarization transfer and there may be a benefit in going to very low fields because there is evidence that these may be more sensitive for hyperpolarized contrast agents 37. The four‐fold higher ^1^H gyromagnetic ratio means that there is a four‐fold lower demand on the gradient system, which, with the availability of high‐quality proton receive coils, makes this an attractive technique for clinical translation.

In conclusion, we have demonstrated a reverse INEPT sequence that allows ^1^H detection of hyperpolarized ^13^C label exchange between injected hyperpolarized [1‐^13^C]pyruvate and the tumor lactate pool. Further incorporation of a spectrally selective ^1^H 90° pulse that flips the magnetization back along the z axis at the end of the reverse INEPT preparation module would allow any ^1^H imaging sequence to be used for signal detection. The sequence is fully compatible with clinical scanners that are already equipped for hyperpolarized ^13^C imaging, where the lower field strengths and consequently longer relaxation times should improve sensitivity.
